# Effects of physical activity distribution patterns on basketball skill acquisition and physical exercise self-efficacy among Chinese male university students: a randomised controlled trial

**DOI:** 10.7717/peerj.21459

**Published:** 2026-08-10

**Authors:** Zhuoxiao Liu, Chengyu Zhou, Zhuo Zeng, Huaibo Yang

**Affiliations:** 1 Strength and Conditioning Training College, Beijing Sports University, Haidian, Beijing, China; 2 School of Physical Education, Shanghai University of Sport, Yangpu, Shanghai, China; 3 Department of Physical Education, Hongshan College of Nanjing University of Finance and Economics, Nanjing, Jiangsu, China

**Keywords:** Basketball, Physical activity patterns, Distributed practice, Technical performance, Physical exercise self-efficacy

## Abstract

**Background:**

As an open-skills sport, basketball not only enhances university students’ physical fitness and specialised techniques but also influences their technical learning efficiency and exercise self-efficacy through patterns of physical activity distribution. However, empirical research on the mechanisms underlying the effects of physical activity distribution patterns on basketball technical performance and self-efficacy remains limited.

**Objective:**

This study aims to investigate the impact of two distinct physical activity patterns (massed *vs.* regularly distributed) on specialised basketball skill acquisition and exercise self-efficacy among Chinese male university students.

**Method:**

A randomised controlled trial design was employed, enrolling 105 male university students aged 18–25 years. The participants were randomly assigned to either a massed training group or a regularly distributed training group. Both interventions lasted 8 weeks, followed by a 2-month follow-up period. Training volume and intensity were maintained at equivalent levels, differing solely in the distribution of practice sessions. Basketball skill acquisition was assessed through shooting accuracy, dribbling agility, and passing precision, whereas psychological efficacy was measured via the Revised Self-Efficacy Scale for Physical Exercise. Generalised estimating equation (GEE) and simple effects analyses were employed to examine between-group and within-group differences, controlling for potential confounding factors.

**Results:**

The findings indicate that the regularly distributed training group demonstrated significantly greater improvements than did the massed training group in shooting (odds ratio (OR) = 2.316, *P* < 0.001), passing (OR = 1.608, *P* < 0.001), and self-efficacy (OR = 2.691, *P* = 0.001), with partial effects persisting two months post-intervention. Both groups demonstrated improvements in dribbling performance, although the differences between groups were not statistically significant.

**Conclusion:**

A regularly distributed physical activity pattern has greater advantages in promoting basketball-specific skills (particularly shooting and passing) and physical exercise self-efficacy. This suggests that basketball training programmes should prioritise optimising practice distribution to increase skill acquisition quality and sustain long-term exercise motivation. Future research may extend to different gender and age groups while incorporating game performance metrics to further validate the efficacy of distributed practice.

## Introduction

In recent years, the health and physical activity levels of university students have drawn considerable attention. Surveys indicate that Chinese university students generally exhibit high participation rates in exercise, with some students’ training frequency meeting the minimum standards recommended by the World Health Organisation. However, the exercise participation patterns of Chinese university students display distinctive structural characteristics: the majority engage in mass physical activity primarily on weekends, whereas the proportion of individuals maintaining regular exercise midweek remains low (Liu, Wang & Zhang, 2021). Exercise patterns remain suboptimal, regular, sustained exercise arrangements are notably absent. Whether this prevailing exercise model facilitates motor skill acquisition, enhances exercise motivation, and increases self-efficacy among Chinese university students constitutes a pressing research question addressed herein. The ‘distribution pattern of physical activity’ may represent a critical factor influencing skill acquisition and self-efficacy enhancement.

For a considerable period, the field of motor learning has debated the relative merits of “massed practice” *versus* “distributed practice”. A substantial body of motor learning research suggests that distributed practice is generally more effective than massed practice in promoting skill consolidation and long-term retention. Experimental studies across various motor tasks and sports skills have consistently demonstrated superior delayed performance following distributed training schedules (Greving & Richter, 2021). Furthermore, distributed practice has been shown to facilitate neural consolidation and reduce fatigue-related performance deterioration (Fuentes-García et al., 2021; Caballero et al., 2024). These findings indicate that the temporal distribution of practice plays a critical role in motor skill acquisition.

From a psychological perspective, exercise self-efficacy plays a critical role in determining individuals’ engagement, persistence, and effort in physical activity (Bandura, 1977). Higher self-efficacy has been consistently associated with more stable exercise behaviour and better intervention adherence among university students (Yu et al., 2024). Therefore, beyond serving as an outcome in its own right, self-efficacy represents a key psychological mechanism through which training interventions may influence the sustainability of physical activity participation.

Basketball, as one of the most popular team sports on Chinese university campuses, holds particular appeal for male undergraduates. Basketball skills—such as dribbling, shooting, passing, and ball control—rely not only in terms of physical attributes (speed, explosiveness, agility) but also heavily in repetitive practice and situational training (shooting on the move, driving to the basket, *etc*.) (Ransone, 2017). Improvements in technical proficiency not only influence match outcomes but also impact participants’ sense of achievement, social recognition, and willingness to continue engaging in sporting activities. This, in turn, may affect their self-efficacy regarding physical exercise and long-term sporting behaviour.

Therefore, treating both basketball skill acquisition and physical exercise self-efficacy as dependent variables holds dual theoretical and practical validity: on the one hand, technical performance reflects the intervention’s direct impact on the “ability-performance” dimension; on the other hand, self-efficacy indicates underlying changes in psychological mechanisms and behavioural maintenance. These two factors mutually influence and reinforce each other (skill enhancement bolsters confidence, whereas heightened self-efficacy promotes higher-quality training commitment) (Han, Li & Zhang, 2022). Incorporating both variables allows simultaneous evaluation of two crucial outcomes: ‘short-term skill acquisition’ and ‘long-term behavioural maintenance’.

However, despite growing international evidence (Bull et al., 2020; Karahan, 2020), empirical studies examining practice distribution in ecologically valid basketball training contexts remain limited, particularly within Chinese university populations. Moreover, few randomised controlled trials have simultaneously investigated the effects of physical activity distribution patterns on both technical performance and psychological outcomes. Therefore, delineating physical activity patterns as ‘massed (two intensive sessions)’ *versus* ‘distributed (five sessions weekly)’ is both theoretically grounded and operationally feasible.

This study aims to compare the effects of regularly distributed physical activity patterns *versus* massed physical activity patterns on the acquisition of specialised basketball skills and self-efficacy in sports participation while examining their maintenance effects during follow-up. Based on previous literature and motor learning theory, the following hypotheses were proposed. H1: participants in the regularly distributed physical activity group will demonstrate greater improvements in basketball technical skills (shooting, passing, and dribbling) over time compared with those in the massed physical activity group. H2: Participants in the regularly distributed physical activity group will show greater improvements in exercise self-efficacy over time than those in the massed physical activity group. H3: The regularly distributed physical activity group will exhibit better maintenance of basketball skills and self-efficacy at follow-up than the massed physical activity group. The overall study design and participant flow are illustrated in Fig. S1.

## Materials and Methods

### Participants

105 participants were randomly assigned to an intervention group (*n* = 53) or a control group (*n* = 52). Baseline demographic information, including age, body mass index (BMI), and prior basketball training experience, was collected through face-to-face interviews conducted by trained research staff. Participants were asked to report their years of basketball practice, which were recorded by the investigators. Prior basketball training experience (months) was included as a covariate in the adjusted models.

The participants were male university students aged between 18 and 25 years. The selection criteria ensured that the participants fully understood the intervention experiment and voluntarily consented to participate. The exclusion criteria were as follows: (1) professional basketball players, all participants had prior recreational basketball experience through physical education classes or informal play. The mean duration of playing experience was 20.75 ± 7.60 months; (2) individuals with current severe sports injuries or inability to participate in training (*e.g.*, significant lower limb injury within the preceding three months); (3) those with diagnosed conditions affecting athletic performance (*e.g.*, cardiovascular, asthma, or neurological disorders); and (4) participants concurrently engaged in other basketball or athletic training programs or planning to compete in other sporting events during the trial period. Written informed consent was obtained from all participants prior to their participation in the study. The study protocol was approved by the Research Ethics Committee of Shanghai University of Sport (Approval No. 102772023RT154).

### Randomisation and blinding

Randomisation was performed by an independent research coordinator using a computer-generated random sequence. Allocation results were sealed in opaque, sequentially numbered envelopes to ensure allocation concealment.

To ensure strict adherence to the blinding protocol, coaches, assessors, independent research coordinators, and statistical analysts were assigned distinct roles, each operating independently. To minimise potential performance and detection bias, maintaining treatment consistency by assigning teaching plans to an experienced coach. Coaches remained unaware of the trial’s purpose and significance while guiding participants through the intervention. Coaches were instructed to follow standardised training protocols and were not involved in outcome assessment or data analysis. Assessors were not informed of participants’ group allocation and were instructed to avoid discussing intervention details with participants. Independent research coordinators completed computer-generated randomisation sequences, which were sealed in opaque envelopes to guarantee allocation concealment. Statistical analysts conducted analyses exclusively on data collected from their assigned intervention, remaining unaware of other intervention components.

Due to the nature of the intervention and the clear difference in training frequency, participants could not be blinded to group allocation. However, outcome assessors and data analysts were blinded to group assignment to minimise potential bias.

All collected data were anonymised and coded prior to analysis. Group labels were masked during statistical analysis and were only revealed after completion of the primary analyses.

### Sample size calculation

The sample size was calculated using G*Power 3.1.9.7 software following the procedures described (Faul et al., 2009). A two-tailed significance level of *α* = 0.05 and a statistical power of 0.80 (1−β) were specified.

The expected effect size (*f* = 0.50) was determined based on recent meta-analytic evidence demonstrating moderate effects of distributed practice on learning and retention outcomes. Specifically, Lee and Genovese (Lee & Genovese, 1988) reported a weighted mean effect size of approximately 0.49 for absolute retention and 0.91 for acquisition in motor skill tasks. For example, a recent meta-analysis reported a standardized mean difference of 0.54 (95% confidence interval (CI) [0.31–0.77]) favoring distributed practice in retention tests (Mawson & Kang, 2025). Contemporary motor learning research consistently supports moderate intervention effects in psychomotor skill acquisition contexts comparable to the present study.

Based on this evidence, a minimum sample size of 41 participants was estimated. Considering an anticipated dropout rate of approximately 15%, the final recruitment target was increased to 96 participants to ensure adequate statistical power.

### Intervention content

Both groups underwent an eight-week basketball-based exercise intervention followed by a two-month follow-up period. The intervention group (distributed physical activity) engaged in 60 min of exercise five times weekly. The control group (massed physical activity) performed 150 min of exercise twice weekly.

Each participant wore a wrist-based heart rate monitor (recorded in real time). All the technical training sessions were conducted within predetermined heart rate zones (70–80% of the maximum heart rate); high-intensity short intervals permitted reaching 85–90% of the maximum heart rate, but the cumulative duration of high-intensity segments within the week must be equal across both groups.

### Intervention design

This study employed a randomised controlled trial design, with the overall research period spanning 18 weeks. The first week comprised baseline testing and adaptation to the core procedures. The subsequent eight weeks constituted the formal intervention period. The postintervention assessment was conducted at week 10, followed by a follow-up assessment at week 18.

To ensure adherence to the intervention protocol, attendance was recorded prior to each training session. All sessions were conducted under the direct supervision of qualified instructors, who monitored participants’ performance (frequency and volume of training) and ensured completion of prescribed training tasks and volumes. Participants missing more than two sessions were excluded from the final analysis. Furthermore, participants wore wrist-based heart rate monitors throughout each experimental intervention to assess exercise intensity and physiological engagement. Heart rate data confirmed participants consistently maintained the prescribed training intensity and did not substantially reduce exercise intensity during sessions. Additionally, all participants provided informed consent and were instructed to adhere to intervention requirements throughout the study.

The intervention and control groups followed identical training protocols for the formal eight-week basketball programme (total weekly practice duration, weekly repetitions per technical skill, sequence and progression rules for training modules, and target heart rate zones). The sole difference between the groups was that the participants were assigned to either a longer massed physical activity pattern or a shorter distributed physical activity pattern. The following represents the total weekly intervention volume, with the subsequent seven weeks repeating the first week’s content from the formal intervention period: (1) shooting: 750 shots per week; (2) dribbling (single-hand dribble, behind-the-back dribble, between-the-legs dribble, spin-back dribble): 100 repetitions per week; (3) passing (chest pass, bounce pass, one-handed pass, *etc*.): 2,500 passes per week; (4) half-court scrimmage: 75 min per week; (5) stretching and rest period: 25 min.

#### Intervention group (distributed physical activity pattern)

The total weekly intervention duration was divided equally into five sessions to determine the duration of each intervention. Each session comprises 5 min of warm-up, 15 min of shooting practice (145–155 shots), 10 min of dribbling practice (20 laps), 10 min of passing practice (500 passes), 15 min of half-court scrimmage, and 5 min of stretching and cool-down. The Regularly Distributed Physical Activity Pattern Intervention Programme shown in Table 1.

**Table 1:** Regularly distributed physical activity pattern intervention programme.

**Intervention varible**	**Project content**	**Detailed requirements**
5-minute warm-up	1. 28*14 m Jog two laps around the basketball court; 2. Hand-held exercise activities.	Warm up thoroughly to activate your body.
15-minute shooting	1. Three-point line stationary shooting:5 spots total, 5 shots per spot, 1 min per set ×5 sets; 2. Three-point line self-rebound shooting: 1 min per set ×3 sets.	1. The spot shooting drill requires completing 25 shots within one minute, with primary focus on shooting accuracy; 2. For self-shooting and rebounding drills, attention should be paid not only to accuracy but also to shooting speed.
10-minute dribbling	Full-court “Z” dribbling, each dribbling technique repeated twice; (Change-of-direction dribble, behind-the-back dribble, between-the-legs dribble, spin dribble.)	When dribbling, run to your position before executing the corresponding action; do not anticipate or delay. Maintain fluidity of movement, lower your centre of gravity, and increase dribbling tempo without compromising accuracy.
10-minute passing	Pair up and practise passing in place. Complete two sets of each passing drill, with 50 repetitions per set, totalling 100 repetitions per passing technique: (Two-handed flat pass, two-handed bounce pass, two-handed overhead pass, one-handed shoulder pass, one-handed bounce pass.)	1. Maintain correct passing technique and swiftly complete the required number of passes per set. 2. Pay attention to distinguishing between each type of pass.
15-minute half-time 5v5 match	Half-court scrimmage, 7 min per session ×2 sets, with a 1-minute interval between sets.	1. Flexibly apply the dribbling, passing and shooting techniques learned; 2. Prevent injuries; 3. Pay attention to team coordination; 4. Maintain concentration throughout the 7 min on court without letting up.
5-Minute stretch and relaxation	1. Jog two laps around the entire pitch; 2. Perform static stretches for the shoulders, neck, and leg ligaments.	Prioritise relaxation activities to alleviate muscle fatigue.

#### Control group (massed physical activity pattern)

The total weekly intervention was divided into two equal portions to determine the volume per session. 13 min warm-up, 37 min shooting (370–380 shots), 25 min dribbling (50 sets), 25 min passing: (1,250 passes), 37 min half-court scrimmage, 13 min stretching and cool-down. The Massed Physical Activity Intervention Programme is shown in Table 2.

**Table 2:** Massed physical activity intervention programme.

**Intervention varible**	**Project content**	**Detailed requirements**
13-minute warm-up	1. Jog two laps around the 400-metre athletics track 2. Hand-held exercise activities.	Warm up thoroughly to activate your body.
37-minute shooting	1. Three-point line stationary shooting: 5 shots per spot, 5 spots, 1 min per set × 10 sets; 2. Three-point line rebound shooting: 1 min per set × 10 sets.	1. The spot shooting drill requires completing 25 shots within one minute, with primary focus on shooting accuracy; 2. For self-shooting and rebounding drills, attention should be paid not only to accuracy but also to shooting speed.
25-minute dribble	Full-court “Z” dribbling, five repetitions of each dribbling technique; (Change-of-direction dribble, behind-the-back dribble, between-the-legs dribble, spin dribble.)	When dribbling, run to your position before executing the corresponding action; do not anticipate or delay. Maintain fluidity of movement, lower your centre of gravity, and increase dribbling tempo without compromising accuracy.
25-minute passing	Pair up and practise passing in place. Complete five sets of each passing drill, with 50 repetitions per set, totalling 250 repetitions per technique: (Two-handed flat pass, two-handed bounce pass, two-handed overhead pass, one-handed shoulder pass, one-handed bounce pass.)	1. Maintain correct passing technique, swiftly complete the required number of passes per set, and ensure distinct differentiation between each passing method. 2. Ensure seamless progression between sets, with rest intervals not exceeding 30 s.
37-minute half-time “5v5” scrimmage	Half-court scrimmage, 7 min per session ×4 sets, with a 2-minute interval between each set.	1. Flexibly apply the dribbling, passing and shooting techniques learned; 2. Prevent injuries; 3. Pay attention to team coordination; 4. Maintain concentration throughout the 7 min on court without letting up.
13-Minute stretch and relaxation	1. Jog two laps around the entire pitch; 2. Perform static stretches for the shoulders, neck, and leg ligaments.	Prioritise relaxation activities to alleviate muscle fatigue.

### Measurement of outcomes

Three methods were employed to evaluate the composite basketball skill score, including shooting accuracy, dribbling agility, and passing precision. Shooting accuracy was assessed through 30 stationary three-point attempts, with each successful shot yielding one point and capping at 30 points. Higher scores indicate superior shooting technique performance (Boddington et al., 2019). Dribbling agility is assessed *via* the ‘dribble deficit’ method, which calculates the ‘dribble deficit’ value by subtracting the time taken to run the same route without the ball from the time taken to dribble the ball. A time of less than 10 s receives a perfect score of 30 points. For every 0.5 s interval, 1 point is deducted, with fractions of 0.5 s rounded to 0.5 s (Scanlan, Tucker & Dalbo, 2018). The passing accuracy is assessed through static passing drills. For different targets—near (three-point line mannequin), medium (half-court line mannequin), and far (backcourt three-point line mannequin)—the following techniques are employed: near target: two-handed chest pass—medium target: two-handed bounce pass—far target: one-handed shoulder pass and two-handed overhead pass. Each position requires execution of all four passing techniques, with five attempts per technique. Hitting the target awards 0.5 points. with a maximum score of 30 points. Higher scores indicate greater passing accuracy.

Self-efficacy was assessed using the Chinese College Students’ Physical Exercise Self-Efficacy Scale (Jiang, Chen & Li, 2018). This 16-item scale employs a five-point Likert scale ranging from ‘1 = Strongly disagree’ to ‘5 = Strongly agree’. Higher scores on each item indicate greater self-efficacy for physical exercise among participants (Jiang, Chen & Li, 2018). The scale demonstrated satisfactory reliability, with a Cronbach’s *α* coefficient of 0.908 (Jiang, Chen & Li, 2018).

### Statistical analysis

This study employed generalised estimating equation (GEE) models to examine the longitudinal effects of two physical activity patterns, namely distributed practice and massed practice, on basketball skill acquisition and physical exercise self-efficacy across three time points: baseline (T0), post-intervention (T1), and two-month follow-up (T2). GEE was selected to account for within-subject correlations arising from repeated measurements.

In the GEE models, participant ID was specified as the subject variable, and measurement time point was specified as the within-subject repeated factor. The outcome variables, including shooting accuracy, dribbling agility, passing precision and physical exercise self-efficacy, were treated as binary variables. A binary logistic model with an exchangeable working correlation structure was used, assuming a constant correlation between repeated measurements within the same participant. Group and time were treated as fixed effects, and the group-by-time interaction was examined to evaluate whether the intervention effects differed between the distributed and massed practice groups over time.

Age, body mass index (BMI), and prior basketball training experience were included as covariates to control for potential confounding effects. Model estimates were reported as regression coefficients (β), odds ratios (ORs), and corresponding 95% confidence intervals (CIs), thereby quantifying the direction and magnitude of the effects of group, time, and their interaction on the outcome variables.

No missing values were identified for the variables included in the final analysis; therefore, multiple imputation was not performed. To assess the robustness of the primary findings, a two-way mixed analysis of variance (ANOVA) was additionally conducted as a sensitivity analysis. The results of this supplementary analysis were broadly consistent with those obtained from the GEE models.

All statistical analyses were performed using R software (version 4.3.1). All statistical tests were two-tailed, and statistical significance was set at *p* < 0.05.

**Figure 1:**
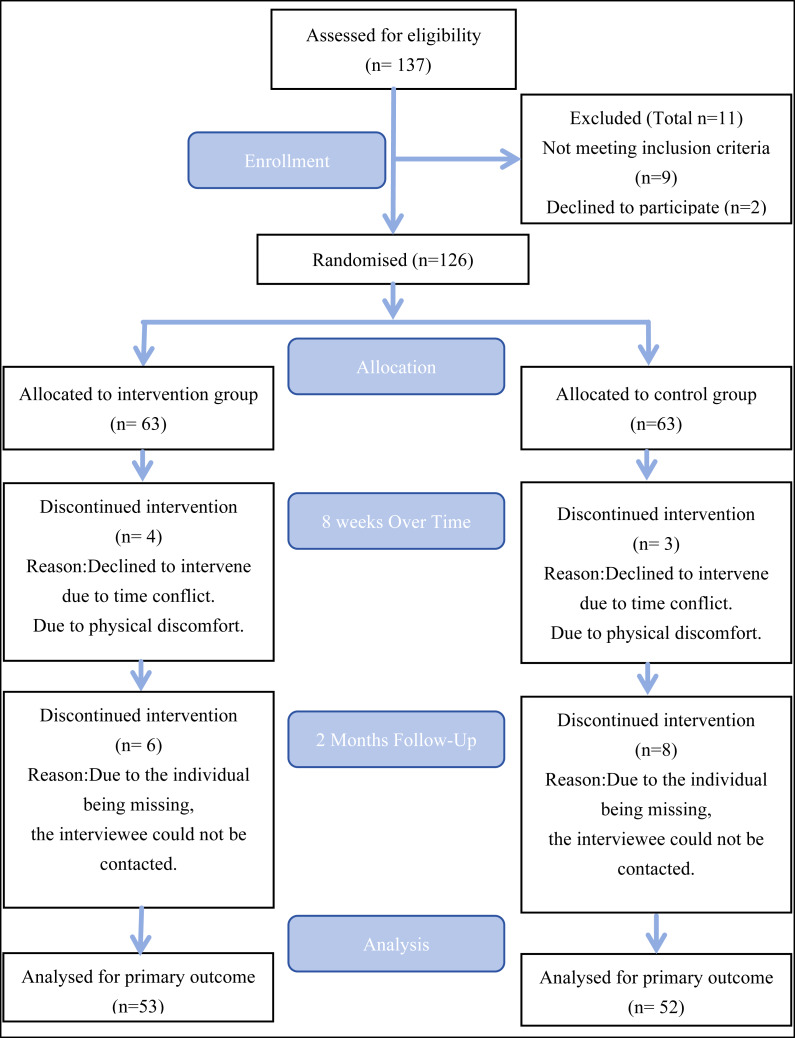
Flow chart of subject selection.

## Results

### Demographic characteristics

This study investigated 105 subjects (control group, *n* = 52; intervention group, *n* = 53), with an average age (years) of 20.93 ± 2.26 years, a mean BMI (kg/m^2^) of 22.82 ± 2.15, and a mean basketball training experience (month) of 20.75 ± 7.60. The participant recruitment flowchart is presented in Fig. 1. Preintervention differences in age (*P* > 0.05), BMI (*P* > 0.05) and basketball training experience (*P* > 0.05) between the control and intervention groups were not statistically significant (see Table 3).

**Table 3:** Participant demographic characteristics.

Varible	*n*	M ± SD	*F*	*t*	*P*	95% CI
Age (years)	105	20.93 ± 2.26	0.381	−0.485	0.628	(−1.119–0.679)
BMI (kg/m^2^)	105	22.82 ± 2.15	0.010	0.439	0.661	(−0.668–1.048)
Basketball training experience (month)	105	20.75 ± 7.60	0.003	−0.144	0.953	(−3.253–2.813)

### Interaction between basketball technical performance and self-efficacy in physical exercise

The generalised estimating equation analyses revealed a significant group-by-time interaction for shooting performance (β = 0.840, *P* < 0.001; OR = 2.316, 95% CI [1.810–2.964]) . The main effect of time was significant (β = −1.000, *P* < 0.001; OR = 0.368, 95% CI [0.257–0.527]), whereas the main effect of group was not significant (β = 0.370, *P* = 0.503) (see Table 4).

**Table 4:** Generalised estimated equation analysis.

Variables	Source	β	SE	*Z*	*P*	OR (95% CI)
Shooting	Time	−1.000	0.183	−5.463	<.001^***^	0.368 (0.257–0.527)
	Group	0.373	0.557	0.670	.503	1.453 (0.488–4.328)
	Time*Group	0.840	0.126	6.674	<.001^***^	2.316 (1.810–2.964)
Dribbling	Time	−0.070	0.259	−0.270	.787	0.932 (0.561-1.549)
	Group	0.270	0.421	0.641	.521	1.310 (0.574–2.989)
	Time*Group	0.340	0.177	1.916	.055	1.405 (0.992–1.989)
Passing	Time	−0.660	0.155	−4.262	<.001^***^	0.517 (0.382–0.700)
	Group	−0.125	0.480	−0.261	.794	0.882 (0.345–2.259)
	Time*Group	0.475	0.108	4.390	<.001^***^	1.608 (1.301–1.988)
Exercise self-efficacy	Time	−0.430	0.427	−1.007	.314	0.651 (0.282–1.502)
	Group	0.170	1.367	0.124	.901	1.185 (0.081–17.275)
	Time*Group	0.990	0.288	3.437	.001^**^	2.691 (1.530–4.733)

**Notes.**

*** Indicates a highly significant difference (*P* < 0.001).

** Indicates a highly significant difference (*P* < 0.01)

No indication indicates no significant difference (*P* > 0.05).

For dribbling performance, the group-by-time interaction was not significant (β = 0.340, *P* = 0.055). Neither the main effect of time (β = −0.070, *P* = 0.787) nor the main effect of group (β = 0.270, *P* = 0.521) reached statistical significance (see Table 4).

For passing performance, a significant group-by-time interaction was observed (β = 0.475, *P* < 0.001; OR = 1.608, 95% CI [1.301–1.988]). The main effect of time was significant (β = −0.660, *P* < 0.001; OR = 0.517, 95% CI [0.382–0.700]), whereas the main effect of group was not significant (β = −0.125, *P* = 0.794) (see Table 4).

With respect to exercise self-efficacy, the main effects of time (β = −0.430, *P* = 0.314) and group (β = 0.170, *P* = 0.901) were not significant. However, a significant group-by-time interaction was identified (β = 0.990, *P* = 0.001; OR = 2.691, 95% CI [1.530–4.733]) (see Table 4).

**Figure 2:**
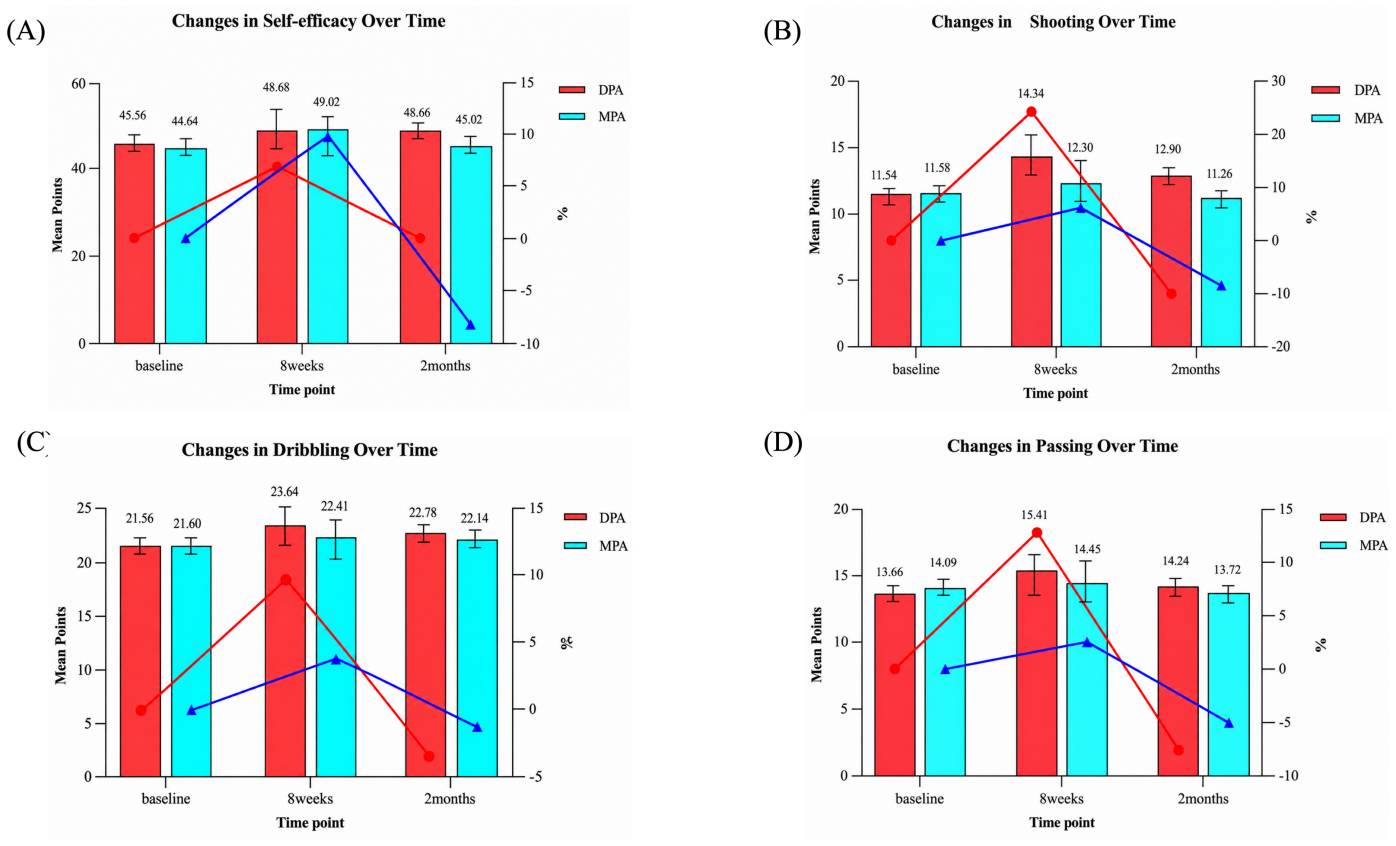
Changes in basketball performance and exercise self-efficacy over time between distributed practice arrangement (DPA) and massed practice arrangement (MPA). (A) Changes in Self-efficacy Over Time. (B) Changes in Shooting Over Time. (C) Changes in Dribbling Over Time. (D) Changes in Passing Over Time. Bars represent mean values with error bars indicating variability. The left Y-axis represents the mean scores shown by the bars, while the right Y-axis represents the percentage changes relative to baseline shown by the lines. DPA, distributed practice arrangement; MPA, massed practice arrangement.

Significant group-by-time interaction effects were observed for shooting, passing, and exercise self-efficacy, whereas no significant interaction was found for dribbling performance (see Fig. 2).

In summary, the results of the generalised estimating equation analysis indicate that the distribution pattern of physical activity significantly positively influences the temporal changes in shooting, passing, and physical exercise self-efficacy among Chinese male university students, whereas no discernible difference in dribbling skills was detected.

### Analysis of differences between groups

Based on the significant group-by-time interactions observed in the GEE analyses, post hoc comparisons were conducted to examine between-group differences at each measurement occasion.

At baseline (T0), no significant differences were observed between the control and intervention groups in shooting (*P* = 0.945), dribbling (*P* = 0.931), passing skills (*P* = 0.409), or self-efficacy for physical exercise (*P* = 0.520), indicating successful randomisation.

At post-intervention (T1), comparisons between the control and intervention groups revealed significantly higher basketball skill scores in the intervention group (shooting *P* < 0.001, dribbling *P* = 0.001, and passing *P* = 0.021) than in the control group. However, no significant difference was observed between the intervention and control groups in self-efficacy for physical exercise.

At the two-month follow-up (T2), comparisons between the control and intervention groups revealed that the intervention group demonstrated significantly higher basketball skill scores (shooting: *P* = 0.005) and self-efficacy (*P* = 0.040) than did the control group. However, no significant differences were observed between the intervention and control groups in dribbling or passing (see Table 5).

**Table 5:** Independent samples *t*-test results at baseline, post-intervention, and two-month follow-up.

	Baseline			8-week intervention outcomes			2 months after intervention		
Variables	M ± SD	*t*	*P*	M ± SD	*t*	*P*	M ± SD	*t*	*P*
Shooting	0.04 ± 0.58	0.07	.945	−2.04 ± 0.54	−3.75	<.001^***^	−1.64 ± 0.58	−2.85	.005^**^
Dribbling	0.04 ± 0.46	0.09	.931	−1.23 ± 0.37	−3.34	.001^**^	−0.64 ± 0.39	−1.66	.101
Passing	0.43 ± 0.52	0.83	.409	−0.96 ± 0.41	−2.34	.021^*^	−0.52 ± 0.48	−1.08	.284
Self-efficacy	−0.92 ± 1.43	−0.65	.520	0.34 ± 1.39	0.24	.808	−2.90 ± 1.40	−2.08	.040^*^

**Notes.**

*** Indicates a highly significant difference (*P* < 0.001).

** Indicates a highly significant difference (*P* < 0.01)

* Indicates a significant difference (*P* < 0.05).

No indication indicates no significant difference (*P* > 0.05).

### Analysis of differences within groups

After an 8-week intervention period (T1), the control group exhibited significant differences in shooting (*P* < 0.001), dribbling (*P* < 0.001), passing technique (*P* = 0.011), and self-efficacy (*P* < 0.001). Within the intervention group, significant improvements were observed in shooting (*P* < 0.001), dribbling (*P* < 0.001), passing technique (*P* < 0.001), and self-efficacy (*P* < 0.001) (see Table 6).

**Table 6:** Basketball skill and self-efficacy outcomes between groups after 8 weeks.

	Massed group (*N* = 52)			Distributed group (*N* = 53)		
Variables	M ± SD	*t*	*P*	M ± SD	*t*	*P*
Shooting	−0.84 ± 1.218	−4.876	<.001^***^	−2.60 ± 1.278	−14.388	<.001^***^
Dribbling	−0.81 ± 1.555	−3.684	<.001^***^	−2.08 ± 2.009	−7.323	<.001^***^
Passing	−0.36 ± 0.964	−2.641	.011^*^	−1.75 ± 1.330	−9.307	<.001^***^
Exercise self-efficacy	−4.38 ± 4.977	−6.223	<.001^***^	−3.12 ± 2.840	−7.768	<.001^***^

**Notes.**

*** Indicates a highly significant difference (*P* < 0.001).

* Indicates a significant difference (*P* < 0.05).

No indication indicates no significant difference (*P* > 0.05).

At the two-month post-intervention time point (T2), no significant differences were observed within the control group for shooting (*P* = 0.613). However, significant differences were noted for dribbling (*P* = 0.018), passing technique (*P* = 0.006), and self-efficacy (*P* = 0.004). Within the intervention group, significant improvements were observed in shooting (*P* < 0.001), dribbling (*P* < 0.001), passing technique (*P* = 0.002), and self-efficacy (*P* < 0.001) (see Table 7).

**Table 7:** Basketball skill and self-efficacy outcomes between groups after two months.

	Massed group (*N* = 52)			Distributed group (*N* = 53)		
Variables	M ± SD	*t*	*P*	M ± SD	*t*	*P*
Shooting	0.10 ± 1.389	0.509	.613	−0.96 ± 1.577	−4.303	<.001^***^
Dribbling	−0.54 ± 1.555	−2.456	.018^*^	−1.22 ± 2.003	−4.307	<.001^***^
Passing	0.37 ± 0.914	2.863	.006^**^	−0.58 ± 1.247	−3.289	.002^**^
Exercise Self-efficacy	−1.12 ± 2.600	−3.046	.004^**^	−3.10 ± 3.190	−6.872	<.001^***^

**Notes.**

*** Indicates a highly significant difference (*P* < 0.001).

** Indicates a highly significant difference (*P* < 0.01)

* Indicates a significant difference (*P* < 0.05).

No indication indicates no significant difference (*P* > 0.05).

## Discussion

This study examined the effects of two physical activity patterns (massed *vs.* regularly distributed) on basketball skill acquisition and exercise self-efficacy among male university students through a randomised controlled trial. The findings indicate that the regularly distributed physical activity pattern significantly enhanced basketball skill acquisition (shooting, passing) and exercise self-efficacy, although no difference in dribbling skills emerged. These findings demonstrate that the two physical activity patterns yield differing degrees of improvement in two specific skills and self-efficacy enhancement.

### The influence of physical activity patterns on basketball skill acquisition

The present findings regarding the influence of physical activity patterns on technical skill acquisition are generally consistent with previous studies (Mawson & Kang, 2025; Czyz, Krzepota & Sadowski, 2024). According to motor learning theory, distributed practice provides greater opportunities for recovery and integration during movement processing and consolidation (Kornmeier, Sosic-Vasic & Joos, 2022). This approach reduces fatigue and skill deterioration associated with single high-dose training sessions, thereby enhancing the efficiency of motor skill acquisition (Kate, Sharma & Singh, 2024; Fahl, Lindner & Zimmermann, 2023). Massed practice, while accumulating a high number of repetitions in a short period, may compromise the quality of each session due to fatigue, thereby undermining long-term skill development (Behrens, Mau-Moeller & Bruhn, 2023). Physiologically, aerobic capacity, muscular strength, and neuromuscular coordination decline within weeks to months of cessation (Marmondi et al., 2025; Mujika & Padilla, 2000). Since basketball skill acquisition relies on foundational physical attributes, progress becomes difficult without adequate strength, speed, endurance, and agility. Although neuroimaging studies have suggested that motor learning is associated with synaptic plasticity, these mechanisms were not directly examined in the present study and therefore remain speculative. Nevertheless, regular and sustained practice may provide favourable conditions for maintaining stable motor performance over time (Gann et al., 2021).

### The influence and retention effects of physical activity patterns on the acquisition of shooting, dribbling and passing techniques

Over time, the effects of the distributed physical activity pattern on shooting, dribbling and passing were more profound than those of the massed physical activity pattern group. They exhibited a slower rate of decline in basketball skill acquisition, with effects persisting for a longer duration.

The large effect size observed during shooting indicates that this task is particularly sensitive to practice distribution. Shooting is a highly refined task that demands consistent movement execution; distributed practice can be more conducive to establishing stable movement patterns and sensorimotor mapping (Ong et al., 2025). This facilitates the formation of more robust memory traces, thereby enhancing the retention rate of motor skills (Brodt et al., 2023). Prolonged intervals between practice sessions can diminish neuromuscular recruitment efficiency, compromising movement stability and precision (Hermosilla-Perona et al., 2025). Second, shooting accuracy is directly correlated with physical fatigue. Research indicates that moderate to severe fatigue significantly reduces shooting accuracy (Li, Zhang & Wang, 2025). Owing to its intensive training load, mass physical activity induces both physical and cognitive fatigue, thereby disrupting the skill consolidation process (Kramár et al., 2012).

In contrast, dribbling performance improved under both practice conditions, and no significant group-by-time interaction was observed. This suggests that both training formats may provide sufficient opportunities for improving this fundamental ball-control skill. The absence of a differential effect between groups may be partly related to differences in practice frequency and session structure. Previous studies have indicated that distributed short-session practice may enhance skill retention compared with massed practice (Kakitani & Kormos, 2024; Middleton, Schuchard & Rawson, 2020; Olivier et al., 2022). However, in the present study, variables such as practice engagement, fatigue, and reactivation patterns were not directly assessed. Therefore, the underlying mechanisms explaining dribbling performance remain speculative and should be examined more systematically in future research.

Passing performance showed moderate interaction effects between group and time. Previous studies have suggested that passing, as a context-dependent skill, is influenced by both technical execution and situational decision-making (Silva, Conte & Clemente, 2020; De Souza et al., 2024). Accordingly, improvements in passing performance may benefit from contextualised and competitive training environments. Passing requires the integration of motor control and tactical decision-making, including pass timing, target selection, and opponent avoidance. Such practice contexts may enhance motivation and engagement, thereby supporting the maintenance of technical proficiency (Maimón, Courel-Ibáñez & Ruíz, 2020). In the present study, the distributed practice model provided opportunities to focus on distinct training scenarios across multiple sessions, which may have facilitated the repeated adaptation of movement strategies (Shamshiri, Fazeli & Nazemzadegan, 2025; Hamilton, Warner & Schwarzer, 2017). In contrast, massed practice may involve multiple scenarios within a single session, potentially limiting opportunities for consolidation and transfer between contexts. Moreover, varied practice conditions have been shown to promote the learning of task-invariant features and improve skill retention (Hamilton, Warner & Schwarzer, 2017; Caballero et al., 2024). However, as contextual adaptation and decision-making processes were not directly assessed in this study, these interpretations remain tentative and warrant further investigation.

Finally, the distributed practice model provides a temporal buffer between the spacing effect and recovery fatigue, enabling athletes to restore attention and movement quality during each session. This mitigates the technical decline or diminished decision-making quality that occurs in massed practice due to fatigue or excessive repetition. Consequently, within the distributed practice group, passing proficiency remains more stable and less prone to deterioration even after the intervention concludes or practice frequency decreases, as training is not confined to highly similar conditions. Although there is currently a lack of specific, long-term (*e.g.*, exceeding 8 weeks plus a maintenance period) empirical research on passing skills, existing experiments on the retention and transfer of jump shot/shooting skills provide strong support (Caballero et al., 2024).

### Influence of physical activity patterns on self-efficacy in sports participation

Self-efficacy demonstrated a large effect size in the interaction, although no between-group or time differences emerged. This finding indicates that changes in self-efficacy over time varied across groups, with the improvement in self-efficacy within the distributed physical activity group being 2.691 times greater than that observed in the massed physical activity pattern. Notably, after an eight-week exercise intervention, no significant difference in self-efficacy was observed between the distributed practice group and the massed practice group. This may be partly explained by several factors. Previous studies have indicated that exercise self-efficacy is strongly influenced by social support, coaching feedback, and peer interaction, in addition to training structure (Hamilton, Warner & Schwarzer, 2017; Bock et al., 2019). When training environments are comparable, similar improvements in self-efficacy may occur across groups (Hampel, Ehlers & Brand, 2023). Moreover, self-efficacy development has been shown to depend on the gradual accumulation of mastery experiences and perceived competence rather than short-term performance changes (Sweet et al., 2014). As both training modes in the present study were associated with improvements in basketball skills, participants in both groups may have experienced comparable gains in perceived competence. However, as psychosocial variables and mastery perceptions were not directly assessed in this study, these explanations remain tentative and warrant further investigation.

### The maintenance effect of physical activity patterns on self-efficacy in sports participation

The follow-up results two months after the intervention revealed that differences re-emerged between the two groups. This finding indicates that either the control group’s self-efficacy decreased relative to two months prior or that the intervention group’s self-efficacy increased relative to two months prior. Several possible explanations may account for this finding. First, regular and sustained physical activity may be associated with favourable perceptions of physical fitness and body image, which have been linked to exercise self-efficacy in previous studies (Zhou, Li & Wang, 2025; Sabiston et al., 2019; Homan & Tylka, 2020). However, these variables were not directly assessed in the present study. Second, distributed practice may facilitate the development of more stable exercise routines, which could support continued engagement in physical activity. Although habit formation has been proposed as a potential mechanism in previous research, it was not formally evaluated in this study. Third, declines in technical performance observed in the massed practice group at follow-up may have influenced perceived competence and confidence in skill execution, which could indirectly affect self-efficacy through changes in perceived competence and mastery experiences. Therefore, these interpretations should be regarded as tentative, and future studies incorporating psychological and behavioural assessments are needed to further clarify the mechanisms underlying self-efficacy maintenance.

The findings have direct practical implications for training arrangements: under conditions where the total training volume is controllable, adopting more regular and distributed physical activity may prove more conducive to enhancing basketball skills and self-efficacy, particularly for shooting skills requiring high stability. When devising training programs, coaches may consider distributing key technical drills across multiple short sessions to ensure the quality of each practice. Concurrently, they should incorporate competitive and situational training to enhance the transfer of passing skills to match situations.

Although decentralised training offers certain advantages in terms of technical performance, the centralised model retains strong practical viability within the context of Chinese university students’ collective culture and course schedules. This suggests that future teaching reforms should gradually introduce regular, sustained training models while respecting local educational structures, thereby balancing feasibility with long-term effectiveness.

### Research limitations and future directions

This study has several limitations. First, the sample was restricted to male university students, and caution is needed when extrapolating the findings to females or other age groups. Second, height and body weight were not separately assessed. Although BMI was used as an indicator of body composition, more detailed anthropometric measurements should be included in future research. Third, self-efficacy data rely on self-reports, which may be subject to recall bias or social desirability bias, thereby undermining the robustness of the conclusions. Fourth, although research indicates that distributed practice has certain advantages in terms of technical skill acquisition, its adherence in real-world settings may be lower than that of massed practice. This is particularly evident among Chinese university students, where academic pressures and constraints on venue availability and scheduling cast doubts on the long-term feasibility of implementing such approaches. Several interpretations proposed in this section are based on theoretical frameworks and previous literature rather than direct measurements in the present study. Therefore, these explanations should be considered exploratory.

Consequently, future research should (1) employ multisource measurement tools (such as objective monitoring combined with questionnaires) to validate the effectiveness of distributed *versus* massed training models across larger samples and diverse populations. Simultaneously, the practical applicability of these models within actual teaching and training contexts should be examined. (2) Incorporating physiological, psychological, and behavioural assessments are needed to further verify the underlying mechanisms. (3) Provides a replicable intervention framework that can be readily applied in other university populations or age groups. Future studies may extend this design by incorporating objective game-performance indicators and competitive outcomes to further examine the ecological validity of distributed practice models.

## Conclusions

This randomised controlled trial demonstrated that, under comparable total training volume and intensity, distributed practice was more effective than massed practice in improving basketball shooting, passing, and exercise self-efficacy among male university students. In contrast, both training models produced similar improvements in dribbling performance, suggesting that dribbling skill acquisition may be less sensitive to practice distribution. These findings indicate that basketball training programmes should prioritise regularly distributed practice to enhance learning quality and long-term skill retention. Coaches and educators may consider organising technical drills across multiple shorter sessions to optimise training outcomes.

## Supplemental Information

10.7717/peerj.21459/supp-1 Supplemental Information 1 Overview of the study design investigating the effects of distributed versus massed practice schedules on basketball-specific skill performance and exercise self-efficacy among college students

10.7717/peerj.21459/supp-2 Supplemental Information 2 Basketball skill performance and exercise self-efficacy scores collected at baseline, post-intervention, and 2-month follow-up across massed and distributed training groups Raw individual-level data for shooting accuracy, dribbling agility, passing precision, and physical exercise self-efficacy scores. Data were collected at three time points for both experimental groups and used for all statistical analyses presented in the manuscript.

## Abbreviations

P: *P* value
BMI: body mass index
OR: Odds ratio
CI: Confidence interval
SE: Standard error
N: Sample size
β: regression coefficient
t: t statistic
Z: Standard normal deviation
F: Joint hypotheses test
M: Mean
SD: Standard deviation

## Additional Information and Declarations

### Data Availability

The following information was supplied regarding data availability: The data is available in the Supplemental File.
